# The top cited clinical research articles on sepsis: a bibliometric analysis

**DOI:** 10.1186/cc11401

**Published:** 2012-06-26

**Authors:** Tianzhu Tao, Xiaohong Zhao, Jingsheng Lou, Lulong Bo, Fei Wang, Jinbao Li, Xiaoming Deng

**Affiliations:** 1 Department of Anesthesiology and Intensive Care, Changhai Hospital, Second Military Medical University, Shanghai 200433, China; 2 Department of Anesthesiology, General Hospital of Jinan Military Area Command, Jinan 250031, Shandong Province, China

## Abstract

**Introduction:**

The objective of this study was to identify and characterize the most highly cited clinical research articles published on sepsis.

**Methods:**

A comprehensive list of citation classics in sepsis was generated by searching the database of Web of Science-Expanded (1970 to present) using keywords 'sepsis' or 'septic shock'. The top 50 cited clinical research papers were retrieved by reading the abstract or full text if needed. Each eligible article was reviewed for basic information, including country of origin, article type, journals, authors, and funding sources.

**Results:**

A total of 2,151 articles were cited more than 100 times; the 50 top-cited clinical articles were published between 1974 and 2008. The number of citations ranged from 372 to 2,932, with a mean of 678 citations per article. These citation classics came from nine countries, of which 26 articles came from the United States. Rush University and the University of Pittsburgh lead the list of classics with six papers each. The 50 top-cited articles were published in 17 journals, with the *New England Journal of Medicine *and *Journal of the American Medical Association *topping the list. The top 50 articles consisted of 21 clinical trials and 29 observational studies.

**Conclusions:**

Our bibliometric analysis provides a historical perspective on the progress of clinical research on sepsis. Articles originating from the United States and published in high-impact journals are most likely to be cited in the field of sepsis research.

## Introduction

Sepsis is a systemic inflammatory response syndrome that occurs during severe infection. It remains a leading cause of death in critically ill patients [1]. Numerous critical care and infectious disease specialists and researchers have focused their efforts on sepsis in an attempt to gain a better understanding of the pathophysiological basis of sepsis or to develop new methods for the diagnosis and treatment of sepsis. Large numbers of articles have been published annually and have given new insights into the mechanism or treatment of sepsis [2].

It is generally accepted that publications represent the central part of a research process. Citation rating is a popular method for evaluating the impact of an investigator or a publication in the scientific community concerned. The frequency of citing has significant implications for authors, journals, institutions and even nations[3]. A remarkable citation history of an author often signifies great honor or recognition in a particular area of research. Although there are obvious disadvantages in assessing the quality of a study simply based on the citation rating, it is widely accepted that this is the best method currently available for judging the merit of a paper or a journal [4]. Citation analysis is also a feasible tool to comprehensively recognize the research advances in the past and future research trends in a specific field.

Clinical research refers to research conducted with human beings, including studies in patient-oriented research, epidemiological and behavioral studies, outcomes and health service research. Clinical study plays a special role in the fight against sepsis because it can provide overwhelming evidence for the treatment and diagnoses of a disease or disorder. Analysis of the most cited articles allows clinical investigators to identify the most popular field of research in sepsis and will give us insights into the characteristics and quality that are required for an article to become widely cited.

Recently, various specialties have attempted to summarize 'citation classics' or the most commonly cited articles in their fields [5-8]. In order to systematically review the citation classics dedicated to sepsis, we conducted the current study to focus exclusively on the 50 top cited clinical articles in an attempt to provide a bibliometric perspective of the progress in sepsis research. We also intended to identify factors contributing to the successful citation such as journals in which the articles were published and related countries.

## Materials and methods

The database of the Institute for Scientific Information (ISI) Web of Science Expanded citation index (1970 to present) was searched using the keyword 'sepsis' or 'septic shock' to identify the citation classics cited more than 400 times. This database includes peer-reviewed publications indexed from more than 10,000 high impact journals worldwide. The 'document type' was applied to limit the format of publications and the type of articles. Papers published as 'article' were selected for further analysis. Each article on the list was reviewed by reading the abstract first and only studies dedicated to clinical research on sepsis were selected for further analysis. The following information was recorded: authors, the number of citations, year of publication, country of origin, institution, journal, funding source, and article type or subfield (for example, randomized controlled trials, observational research). We also calculated the publication output on sepsis by normalizing the total number of articles to the gross domestic product (GDP) per capita of the included countries. The information of GDP per capita was retrieved from the website of the World Bank. All electronic searches were conducted on 13 March 2012. Correlation analysis between GDP per capita and the total number of articles on sepsis was conducted using GraphPad Prism 5.0 software.

## Results

A total of 67,558 papers were identified in the initial search for the period from 1970 to present, with 50,192 published as 'article' and 4,748 classified as 'review'. Among them, 2,151 articles were cited more than 100 times.

Of the top 50 cited clinical trials, the mean number of citations was 678 (range 372 to 2,932) and six papers were cited more than 1,000 times (Table 1). These articles were published between 1974 and 2008, of which about 50% were published after 1995 (Table 2).

**Table 1:** The top 50 cited clinical trials on sepsis.

Ratings	Article	No. of citations
**1**	Bernard GR, Vincent JL, Laterre P, LaRosa SP, Dhainaut JF, Lopez-Rodriguez A, Steingrub JS, Garber GE, Helterbrand JD, Ely EW, Fisher CJ Jr: **Efficacy and safety of recombinant human activated protein C for severe sepsis**. *New England Journal of Medicine *2001, **344**:699-709.	**2932**
**2**	Rivers E, Nguyen B, Havstad S, Ressler J, Muzzin A, Knoblich B, Peterson E, Tomlanovich M: Early Goal-Directed Therapy C: **Early goal-directed therapy in the treatment of severe sepsis and septic shock**. *New England Journal of Medicine *2001, **345**:1368-1377.	**2538**
**3**	Angus DC, Linde-Zwirble WT, Lidicker J, Clermont G, Carcillo J, Pinsky MR: **Epidemiology of severe sepsis in the United States: Analysis of incidence, outcome, and associated costs of care**. *Critical Care Medicine *2001, **29**(7):1303-1310.	**2158**
**4**	Martin GS, Mannino DM, Eaton S, Moss M: **The epidemiology of sepsis in the United States from 1979 through 2000**. *New England Journal of Medicine *2003, **348**:1546-1554.	**1551**
**5**	Annane D, Sebille V, Charpentier C, Bollaert PE, François B, Korach JM, Capellier G, Cohen Y, Azoulay E, Troché G, Chaumet-Riffaud P, Bellissant E: **Effect of treatment with low doses of hydrocortisone and fludrocortisone on mortality in patients with septic shock**. *JAMA-Journal of the American Medical Association *2002, **288**:862-871.	**1227**
**6**	Ziegler EJ, Fisher CJ, Sprung CL, Straube RC, Sadoff JC, Foulke GE, Wortel CH, Fink MP, Dellinger RP, Teng NN: **Treatment of gram-negative bacteremia and septic shock with HA-1a human monoclonal-antibody against endotoxin - a randomized, double-blind, placebo-controlled trial**. *New England Journal of Medicine *1991, **324**:429-436.	**1152**
**7**	Bone RC, Fisher CJ, Clemmer TP, Slotman GJ, Metz CA, Balk RA: **A controlled clinical-trial of high-dose methylprednisolone in the treatment of severe sepsis and septic shock**. *New England Journal of Medicine *1987, **317**:653-658.	**902**
**8**	Rangelfrausto MS, Pittet D, Costigan M, Hwang T, Davis CS, Wenzel RP: **The natural-history of the systemic inflammatory response syndrome (SIRS) - a prospective-study**. *JAMA-Journal of the American Medical Association *1995, **273**:117-123.	**827**
**9**	Brunkhorst FM, Engel C, Bloos F, Meier-Hellmann A, Ragaller M, Weiler N, Moerer O, Gruendling M, Oppert M, Grond S, Olthoff D, Jaschinski U, John S, Rossaint R, Welte T, Schaefer M, Kern P, Kuhnt E, Kiehntopf M, Hartog C, Natanson C, Loeffler M, Reinhart K: **Intensive insulin therapy and pentastarch resuscitation in severe sepsis**. *New England Journal of Medicine *2008, **358**:125-139.	**738**
**10**	Casey LC, Balk RA, Bone RC: **Plasma cytokine and endotoxin levels correlate with survival in patients with the sepsis syndrome**. *Annals of Internal Medicine *1993, **119**:771-778.	**729**
**11**	Assicot M, Gendrel D, Carsin H, Raymond J, Guilbaud J, Bohuon C: **High serum procalcitonin concentrations in patients with sepsis and infection**. *Lancet *1993, **341**:515-518.	**693**
**12**	Parker MM, Shelhamer JH, Bacharach SL, Green MV, Natanson C, Frederick TM, Damske BA, Parrillo JE: **Profound but reversible myocardial depression in patients with septic shock**. *Annals of Internal Medicine *1984, **100**:483-490.	**655**
**13**	Kudsk KA, Croce MA, Fabian TC, Minard G, Tolley EA, Poret HA, Kuhl MR, Brown RO: **Enteral versus parenteral-feeding - effects on septic morbidity after blunt and penetrating abdominal-trauma**. *Annals of Surgery *1992, **215**:503-513.	**650**
**14**	Fisher CJ, Agosti JM, Opal SM, Lowry SF, Balk RA, Sadoff JC, Abraham E, Schein RM, Benjamin E: **Treatment of septic shock with the tumor necrosis factor receptor:Fc fusion protein**. *New England Journal of Medicine *1996, **334**:1697-1702.	**649**
**15**	Hack CE, Degroot ER, Feltbersma RJF, Nuijens JH, Strack Van Schijndel RJ, Eerenberg-Belmer AJ, Thijs LG, Aarden LA:**Increased plasma-levels of interleukin-6 in sepsis**. *Blood *1989, **74**:1704-1710.	**643**
**16**	Kumar A, Roberts D, Wood KE, Light B, Parrillo JE, Sharma S, Suppes R, Feinstein D, Zanotti S, Taiberg L, Gurka D, Kumar A, Cheang M: **Duration of hypotension before initiation of effective antimicrobial therapy is the critical determinant of survival in human septic shock**. *Critical Care Medicine *2006, **34**(6):1589-1596.	**598**
**17**	Fisher CJ, Dhainaut JFA, Opal SM, Pribble JP, Balk RA, Slotman GJ, Iberti TJ, Rackow EC, Shapiro MJ, Greenman RL: **Recombinant human interleukin-1 receptor antagonist in the treatment of patients with sepsis syndrome - results from a randomized, double-blind, placebo-controlled trial**. *JAMA-Journal of the American Medical Association *1994, **271**(23):1836-1843.	**591**
**18**	Greenman RL, Schein RMH, Martin MA, Wenzel RP, MacIntyre NR, Emmanuel G, Chmel H, Kohler RB, McCarthy M, Plouffe J: **A controlled clinical-trial of E5 murine monoclonal IgM antibody to endotoxin in the treatment of gram-negative sepsis**. *JAMA-Journal of the American Medical Association *1991, **266**:1097-1102.	**564**
**19**	Warren BL, Eid A, Singer P, Pillay SS, Carl P, Novak I, Chalupa P, Atherstone A, Pénzes I, Kübler A, Knaub S, Keinecke HO, Heinrichs H, Schindel F, Juers M, Bone RC, Opal SM: **High-dose a randomized antithrombin III in severe sepsis - A randomized controlled trial**. *JAMA-Journal of the American Medical Association *2001, **286**:1869-1878.	**552**
**20**	Docke WD, Randow F, Syrbe U, Krausch D, Asadullah K, Reinke P, Volk HD, Kox W: **Monocyte deactivation in septic patients: Restoration by IFN-gamma treatment**. *Nature Medicine *1997, **3**:678-681.	**510**
**21**	Pinsky MR, Vincent JL, Deviere J, Alegre M, Kahn RJ, Dupont E: **Serum cytokine levels in human septic shock - relation to multiple-system organ failure and mortality**. *Chest *1993, **103**:565-575.	**506**
**22**	Ochoa JB, Udekwu AO, Billiar TR, Curran RD, Cerra FB, Simmons RL, Peitzman AB: **Nitrogen-oxide levels in patients after trauma and during sepsis**. *Annals of Surgery *1991, **214**:621-626.	**505**
**23**	Brunbuisson C, Doyon F, Carlet J, Dellamonica P, Gouin F, Lepoutre A, Mercier JC, Offenstadt G, Régnier B: **Incidence, risk-factors, and outcome of severe sepsis and septic shock in adults - a multicenter prospective-study in intensive-care units**. *JAMA-Journal of the American Medical Association *1995, **274**:968-974.	**498**
**24**	Abraham E, Wunderink R, Silverman H, Perl TM, Nasraway S, Levy H, Bone R, Wenzel RP, Balk R, Allred R: **Efficacy and safety of monoclonal-antibody to human tumor-necrosis-factor-alpha in patients with sepsis syndrome - a randomized, controlled, double-blind, multicenter clinical-trial**. *JAMA-Journal of the American Medical Association *1995, **273**:934-941.	**491**
**25**	Stoll BJ, Hansen N, Fanaroff AA, Wright LL, Carlo WA, Ehrenkranz RA, Lemons JA, Donovan EF, Stark AR, Tyson JE, Oh W, Bauer CR, Korones SB, Shankaran S, Laptook AR, Stevenson DK, Papile LA, Poole WK: **Late-onset sepsis in very low birth weight neonates: The experience of the NICHD Neonatal Research Network**. *Pediatrics *2002, **110**:285-291.	**490**
**26**	Calandra T, Baumgartner JD, Grau **GE**, Wu MM, Lambert PH, Schellekens J, Verhoef J, Glauser MP: **Prognostic values of tumor-necrosis-factor cachectin, interleukin-1, interferon-alpha, and interferon-gamma in the serum of patients with septic shock**. *Journal of Infectious Diseases *1990, **161**:982-987.	**473**
**27**	Damas P, Ledoux D, Nys M, Vrindts Y, De Groote D, Franchimont P, Lamy M: **Cytokine serum level during severe sepsis in human IL-6 as a marker of severity**. *Annals of Surgery *1992, **215**:356-362.	**465**
**28**	Hotchkiss RS, Swanson PE, Freeman BD, Tinsley KW, Cobb JP, Matuschak GM, Buchman TG, Karl IE: **Apoptotic cell death in patients with sepsis, shock, and multiple organ dysfunction**. *Critical Care Medicine *1999, **27**:1230-1251.	**463**
**29**	Sprung CL, Caralis PV, Marcial EH, Tinsley KW, Cobb JP, Matuschak GM, Buchman TG, Karl IE: **The effects of high-dose corticosteroids in patients with septic shock - a prospective, controlled-study**. *New England Journal of Medicine *1984, **311**:1137-1143.	**461**
**30**	Danner RL, Elin RJ, Hosseini JM, Wesley RA, Reilly JM, Parillo JE: **Endotoxemia in human septic shock**. *Chest *1991, **99**:169-175.	**458**
**31**	Sprung CL, Annane D, Keh D, Moreno R, Singer M, Freivogel K, Weiss YG, Benbenishty J, Kalenka A, Forst H, Laterre PF, Reinhart K, Cuthbertson BH, Payen D, Briegel J: **Hydrocortisone therapy for patients with septic shock**. *New England Journal of Medicine *2008, **358**:111-124.	**458**
**32**	Meakins JL, Pietsch JB, Bubenick O, Kelly R, Rode H, Gordon J, MacLean LD: **Delayed-hypersensitivity - indicator of acquired failure of host defenses in sepsis and trauma**. *Annals of Surgery *1977, **186**:241-250.	**449**
**33**	Annane D, Sebille V, Troche G, Raphaël JC, Gajdos P, Bellissant E: **A 3-level prognostic classification in septic shock based on cortisol levels and cortisol response to corticotropin**. *JAMA-Journal of the American Medical Association *2000, **283**:1038-1045.	**444**
**34**	Suter PM, Suter S, Girardin E, Roux-Lombard P, Grau GE, Dayer JM: **High bronchoalveolar levels of tumor-necrosis-factor and its inhibitors, interleukin-1, interferon, and elastase, in patients with adult respiratory-distress syndrome after trauma, shock, or sepsis**. *American Review of Respiratory Disease *1992, **145**:1016-1022.	**443**
**35**	Briegel J, Forst H, Haller M, Schelling G, Kilger E, Kuprat G, Hemmer B, Hummel T, Lenhart A, Heyduck M, Stoll C, Peter K: **Stress doses of hydrocortisone reverse hyperdynamic septic shock: A prospective, randomized, double-blind, single-center study**. *Critical Care Medicine *1999, **27**:723-732.	**426**
**36**	Bollaert PE, Charpentier C, Levy B, Debouverie M, Audibert G, Larcan A: **Reversal of late septic shock with supraphysiologic doses of hydrocortisone**. *Critical Care Medicine *1998, **26**:645-650.	**425**
**37**	Munoz C, Carlet J, Fitting C, Misset B, Blériot JP, Cavaillon JM: **Dysregulation of invitro cytokine production by monocytes during sepsis**. *Journal of Clinical Investigation *1991, **88**:1747-1754.	**424**
**38**	Vincent JL, Sakr Y, Sprung CL, Ranieri VM, Reinhart K, Gerlach H, Moreno R, Carlet J, Le Gall JR, Payen D: **Sepsis in European intensive care units: Results of the SOAP study**. *Critical Care Medicine *2006, **34**:344-353.	**415**
**39**	Fourrier F, Chopin C, Goudemand J, Hendrycx S, Caron C, Rime A, Marey A, Lestavel P: **Septic shock, multiple organ failure, and disseminated intravascular coagulation - compared patterns of antithrombin-III, protein-C, and protein-S deficiencies**. *Chest *1992, **101**:816-823.	**414**
**40**	Mira JP, Cariou A, Grall F, Delclaux C, Losser MR, Heshmati F, Cheval C, Monchi M, Teboul JL, Riché F, Leleu G, Arbibe L, Mignon A, Delpech M, Dhainaut JF: **Association of TNF2, a TNF-alpha promoter polymorphism, with septic shock susceptibility and mortality - A multicenter study**. *JAMA-Journal of the American Medical Association *1999, **282**:561-568.	**412**
**41**	Marik PE, Sibbald WJ: **Effect of stored-blood transfusion on oxygen delivery in patients with sepsis**. *JAMA-Journal of the American Medical Association *1993, **269**:3024-3029.	**411**
**42**	Petros A, Lamb G, Leone A, Moncada S, Bennett D, Vallance P: **Effects of a nitric-oxide synthase inhibitor in humans with septic shock**. *Cardiovascular Research *1994, **28**:34-39.	**410**
**43**	Clowes GHA, George BC, Villee CA, Saravis CA: **Muscle proteolysis induced by a circulating peptide in patients with sepsis or trauma**. *New England Journal of Medicine *1983, **308**:545-552.	**407**
**44**	Abraham E, Laterre P, Garg R, Levy H, Talwar D, Trzaskoma BL, François B, Guy JS, Brückmann M, Rea-Neto A, Rossaint R, Perrotin D, Sablotzki A, Arkins N, Utterback BG, Macias WL: **Drotrecogin alfa (activated) for adults with severe sepsis and a low risk of death**. *New England Journal of Medicine *2005, **353**:1332-1341.	**401**
**45**	Abraham E, Reinhart K, Opal S, Demeyer I, Doig C, Rodriguez AL, Beale R, Svoboda P, Laterre PF, Simon S, Light B, Spapen H, Stone J, Seibert A, Peckelsen C, De Deyne C, Postier R, Pettilä V, Artigas A, Percell SR, Shu V, Zwingelstein C, Tobias J, Poole L, Stolzenbach JC, Creasey AA:**Efficacy and safety of tifacogin (recombinant tissue factor pathway inhibitor) in severe sepsis - A randomized controlled trial**. *JAMA-Journal of the American Medical Association *2003, **290**:238-247.	**395**
**46**	Noone P, Parsons TMC, Pattison JR, Slack RC, Garfield-Davies D, Hughes K: **Experience in monitoring gentamicin therapy during treatment of serious gram-negative sepsis**. *British Medical Journal *1974, **1**:477-481.	**384**
**47**	Landry DW, Levin HR, Gallant EM, Ashton RC Jr, Seo S, D'Alessandro D, Oz MC, Oliver JA: **Vasopressin deficiency contributes to the vasodilation of septic shock**. *Circulation *1997, **95**:1122-1125.	**383**
**48**	Askanazi J, Carpentier YA, Elwyn DH, Nordenström J, Jeevanandam M, Rosenbaum SH, Gump FE, Kinney JM: **Influence of total parenteral-nutrition on fuel utilization in injury and sepsis**. *Annals of Surgery *1980, **191**:40-46.	**381**
**49**	Michard F, Boussat S, Chemla D, Anguel N, Mercat A, Lecarpentier Y, Richard C, Pinsky MR, Teboul JL: **Relation between respiratory changes in arterial pulse pressure and fluid responsiveness in septic patients with acute circulatory failure**. *American Journal of Respiratory and Critical Care Medicine *2000, **162**:134-138.	**372**
**50**	Lorenz E, Mira JP, Frees KL, Schwartz DA: **Relevance of mutations in the TLR4 receptor in patients with gram-negative septic shock**. *Archives of Internal Medicine 2002***, 162***:1028-1032*.	**372**

**Table 2:** Frequency distribution showing publication years of the 50 top-cited articles.

Period	Number of articles	Mean number of citations
1970 to 1984	6	455
1985 to 1989	3	773
1990 to 1994	16	556
1995 to 1999	10	508
2000 to 2004	10	1593
2005 to present	5	522

The 50 top cited articles originated from nine countries, with the United States (26) and France (8) being the most prolific (Table 3). Given some articles were authored with multiple sources of origin, especially those in the form of international research collaborations, the country of origin was defined by the address of the corresponding author. The leading institutions are shown in Table 4. Rush University and the University of Pittsburgh were found to be the most productive institutions, with six articles each. There was a weak correlation between the GDP per capita and the numbers of articles on sepsis related to the nine countries (r^2 ^= 0.02).

**Table 3:** Countries of origin of the top 50 cited articles on sepsis.

Country	Number of articles	GDP per capita (US Dollar)
US	26	47,390
France	8	42,390
Germany	4	43,110
Canada	3	43,270
Switzerland	3	71,530
UK	2	38,370
Belgium	2	45,910
Netherlands	1	49,050
Chile	1	10,120

**Table 4:** Institutions of origin with three or more top-cited articles on sepsis.

Rank	Institution	Number of articles
1	Rush University(Chicago)	6
2	University of Pittsburgh(Pennsylvania)	6
3	Brown University(Rhode Island)	5
4	University of Miami (Florida)	5
5	Cleveland Clinic Foundation(Cleveland)	3
6	Erasme University Hospital(Brussels)	3

The 50 top cited clinical articles were published in 17 journals, predominantly in *New England Journal of Medicine *(*n *= 11) and *Journal of the American Medical Association *(*n *= 11), followed by *Critical Care Medicine *(*n *= 6) (Table 5). Table 6 presents a list of the most productive authors, indicating that Sprung CL authored six articles, followed by Abraham E, Bone RC, Fisher CJ and Vincent JL.

**Table 5:** Journals in which the top 50 cited articles on sepsis were published.

Rank	Journal	Number of articles	Impact factor(2010)
1	New England Journal of Medicine	11	53.48
2	Journal of the American Medical Association	11	30.01
3	Annals of Internal Medicine	2	16.72
4	Annals of Surgery	5	7.47
5	Chest	3	6.52
6	Critical Care Medicine	6	6.25

**Table 6:** The most common authors of top 50 cited clinical research studies on sepsis.

Author	Number of articles	First author	Second author	Last author
Sprung, CL.	6	2	0	0
Abraham, E	5	3	0	0
Fisher, CJ	5	2	2	1
Bone, RC	5	1	0	1
Balk, RA	5	0	1	1
Vincent, JL	4	1	2	0
Annane, D	3	2	0	0
Levy, H	3	0	0	0

Subgroup analysis of the top cited studies indicated that biomarkers (10, 11, 15, 21, 22, 26, 27, 30, 34, 40 in Table 1), immunology (6, 14, 17, 18, 20, 24, 28, 32, 37, 45 in Table 1), hemodynamics (2, 9, 12, 16, 19, 39, 47, 49 in Table 1), steroids (5, 7, 29, 31, 33, 35, 36 in Table 1) and epidemiology (3, 4, 8, 23, 25, 38, 50 in Table 1) were the most popular topics.

Of the 50 top cited articles, 21 reported clinical trials, of which 19 were designed as randomized controlled trials (RCT) and 29 reported observational studies. Among these original research papers, 15 were funded by public foundations, 12 received support from commercial companies, six were supported by both, and for the remaining 17 the funding source was not specified (Table 7). Specifically, five studies received grants from the National Institutes of Health (NIH). Eight of the 19 RCTs reported supportive interventions and 11 studies showed detrimental or neutral effects [See Additional file 1].

**Table 7:** Funding source of the top 50 cited clinical research studies.

Fund	Number
Public	15
Industry	12
Both	6
Not specified	17
Total	50

## Discussion

The present study summarizes several features of influential articles in sepsis research during the past 40 years by means of a literature review. It was found that 26 (52%) of the 50 articles were from the United States. The country dominance is also found in other clinical disciplines including Urology [5], Orthopedic Surgery [6], Critical Care Medicine [7], General Surgery [8], Emergency Medicine [9] and Anesthesia [10]. The underlying reasons might be due to its large population of senior researchers and adequate research budgets for scientific investigation. It is generally presumed that the correlation between the research output and GDP should be an important issue in bibliometric analysis. But we found that the correlation between the GDP per capita and the total number of articles published on sepsis was weak in the related nine countries. This might be due to the limited number of retrieved articles and included countries. In addition, it was found that American authors tended to cite local papers [11] and US reviewers had a significant preference to accepting papers written by native researchers [12]. Our study found that most of the classics were published in high-impact journals, which is consistent with the result of other reviews, supporting the well known paradigm that top cited articles are often published in journals topping the impact factor list, which in turn maintains the high impact factor of these journals [13].

Financial support from public foundations or commercial companies has evolved over time in response to changes in professional codes, laws and markets[14]. Public funds have given a great push to the development of medical research and public health. In our study, 15 papers reported funding support from public institutions or national foundations. Industry-funded science has been widely debated because of the susceptibility to various kinds of biases. Nevertheless, it has played and will continue to play a critical role in the research process [15]. Our review shows that a total of 18 research projects received grants from ommercial companies. A remarkable thing should be considered that some papers, especially the old ones, might not have fully reported their financial conflicts.

Clinical research has bridged the gap between basic science and improved human health and is heavily weighted towards biomedical science. In the past 40 years, clinical studies have tested large numbers of therapeutic agents and provided insights into the pathophysiological basis of sepsis. The list of the top cited articles shows some interesting trends and pinpoints major advances in sepsis research. Sepsis has been defined as hyper-inflammation and excessive activation of the immune system characterized by a prolonged cytokine storm. Therefore, many studies have targeted certain cytokines highly expressed in sepsis for therapeutic or diagnostic application. IL-6 and TNF-α appeared to be good markers for predicting severity and prognosis of sepsis. However, clinical trials have failed to demonstrate promising results using antibody blockade to these cytokines, for sepsis initiates much more complex immunologic responses. Glucocorticoids have been another hot topic in the field of sepsis research. Owing to the positive effect on the sensitivity of vascular smooth muscle to catecholamines, steroid has been considered as a promising agent in septic shock patients. Currently, the use of glucocorticoid cannot be recommended as the standard of care, but it is feasible in the course for patients with septic shock that does not respond to conventional measures.

Of the 19 RCTs, eight demonstrated promising and supportive interventions [See Additional file 1]. Considering that RCT-based studies provide greater quality evidence than other study designs, trials with promising results often encourage clinicians to apply these interventions. Unfortunately, successful measures for sepsis treatment seem difficult to achieve; in the top cited 50 articles, 11 of the 19 RCTs have proved to be non-efficacious. Reasons related to the negative results include the reporting quality of these studies, small sample size or heterogeneity of the ICU patient population[16].

Our review has several limitations. First, we elected to limit our research to 'sepsis' or 'septic shock' in the topic field, which may miss some citations related to our analysis such as those indexed with 'LPS'. In addition, our search via the Web of Science expanded database from 1970 to present is quite sensitive and papers published before were excluded, so it is likely that some true 'classic' articles were missed in this review.

Another limitation is the inherent bias of the citation analysis [17]. Total cites of an article accumulate over time which means that older publications would definitely receive more citations than new ones. Recent publications with a short span of time to generate citation rates are possibly underestimated with respect to their impact. In addition, oriented or biased citing, including self-citation, in-house, or negative citation (bias towards potential negative credits) is also a problem that should not be ignored [18]. An important thing to be kept in mind is that impact factor or citation analysis is not an index to evaluate the quality of scientific research, but rather a measure of recognition. In other words, the number of citations of an article should not be considered equivalent to its importance.

## Conclusions

Our bibliometric analysis provides a historical perspective on the progress in clinical sepsis research in the past 40 years. Papers originated from the US and published in high-impact journals are most likely to be cited in the field of sepsis research.

## Key messages

• The mean number of citations per article was 678 and six papers were cited more than 1,000 times.

• The US is responsible for the most contributions to clinical studies on sepsis.

• The top cited 50 articles on sepsis were published in 17 journals, led by the *New England Journal of Medicine *(*n *= 11) and the *Journal of the American Medical Association *(*n *= 11), followed by *Critical Care Medicine *(*n *= 6).

• About half of research papers received funding support from public institutions or national foundations.

• Nineteen articles were designed as RCTs, among which eight papers reported promising results.

## Abbreviations

IF: impact factor; IL: interleukin; LPS: lipopolysaccharide; NIH: National Institutes of Health; RCT: randomized controlled trial; TNF: tumor necrosis factor.

## Competing interests

The authors declare that they have no competing interests.

## Authors' contributions

TZT, XHZ and JSL contributed equally to this article. They all participated in the study design, data collection and also drafting the manuscript. LLB and FW helped in the design of the study and analyzed the data. Both XMD and JBL designed this study, supervised the data collection and wrote this article. All authors read and approved the final manuscript.

## Supplementary Material

Additional file 1 **Therapy or agents tested in RCTs on sepsis**. Nineteen studies among the citation classics were designed as RCTs. Treatments tested in eight articles were found to be promising or supportive interventions. Click here for file
