# Supplementary material for: The top cited clinical research articles on sepsis: a bibliometric analysis
Source: Crit Care. 2012 Jun 26;16(3):R110. doi: 10.1186/cc11401 (PMC3580668; doi:10.1186/cc11401)
Supplement: Additional file 1 — Therapy or agents tested in RCTs on sepsis. Nineteen studies among the citation classics were designed as RCTs. Treatments tested in eight articles were found to be promising or supportive interventions. [file cc11401-S1.DOCX]

**Additional file 1 Therapy or agents tested in RCTs on sepsis**

|  | Therapy | Participants | Interventions | Findings | Ref No. in Table 1 |
| --- | --- | --- | --- | --- | --- |
| Positive | recombinant human activated protein C (Drotrecogin alfa) | patients with systemic inflammation and organ failure due to acute infection | drotrecogin alfa activated (24 microg per kilogram of body weight per hour) for a total duration of 96 hours or placebo | recombinant human activated protein C reduces mortality in patients with severe sepsis and may be associated with an increased risk of bleeding | **1** |
|  | early goal-directed therapy | patients with severe sepsis or septic shock | six hours of early goal-directed therapy or standard therapy before admission to the intensive care unit | early goal-directed therapy provides significant benefits with respect to outcome in patients with severe sepsis and septic shock | **2** |
|  | low doses of hydrocortisone and fludrocortisone | patients who fulfilled usual criteria for septic shock | hydrocortisone (50-mg intravenous bolus every 6 hours) and fludrocortisone (50- micro g tablet once daily) or matching placebos | a 7-day treatment with low doses of hydrocortisone and fludrocortisone significantly reduced the risk of death in patients with septic shock and relative adrenal insufficiency without increasing adverse events | **5** |
|  | HA-1A human monoclonal antibody against endotoxin | patients with sepsis and a presumed diagnosis of gram-negative infection | a single 100-mg intravenous dose of HA-1A (in 3.5 g of albumin) or placebo (3.5 g of albumin) | HA-1A is safe and effective for the treatment of patients with sepsis and gram-negative bacteremia | **6** |
|  | early enteral feeding | patients with an abdominal trauma index of at least 15 w | enteral or parenteral feeding within 24 hours with indentical food formulas | there is a significantly lower incidence of morbidity in patients fed enterally after blunt and penetrating trauma, with most of the significant changes occurring in the more severely injured patients. | **13** |
|  | E5 murine monoclonal IgM antibody to endotoxin | patients with signs of gram-negative infection and a systemic septic response | 2 mg/kg of a murine monoclonal antibody directed against gram-negative endotoxin (E5) or placebo | treatment with E5 antiendotoxin antibody reduces mortality and enhances the resolution of organ failure among patients with gram-negative sepsis who are not in shock when treated | **18** |
|  | High-dose antithrombin III | severe sepsis patients with a high risk of death | high-dose antithrombin III (30,000 IU intravenously over the period of 4 days) or placebo | treatment with high-dose antithrombin III may increase survival time up to 90 days in patients with severe sepsis and high risk of death. This benefit may even be stronger when concomitant heparin is avoided | **19** |
|  | supraphysiologic doses of hydrocortisone | patients with septic shock requiring catecholamine for >48 hrs | Patients received either hydrocortisone (100 mg iv three times daily for 5 days) or matching placebo | Administration of modest doses of hydrocortisone in the setting of pressor-dependent septic shock for a mean of >96 hrs resulted in a significant improvement in hemodynamics and a beneficial effect on survival | **35** |
|  | high-dose methylprednisolone | patients with clinical suspicion of infection plus the presence of fever or hypothermia , tachypnea , tachycardia, and the presence of one of indications of organ dysfunction | methylprednisolone sodium succinate (30 mg per kilogram of body weight) or placebo | high-dose corticosteroids provides no benefit in the treatment of severe sepsis and septic shock | **7** |
|  | intensive insulin therapy and pentastarch resuscitation | patients with severe sepsis | intensive insulin therapy to maintain euglycemia or conventional insulin therapy and either 10% pentastarch, a low-molecular-weight hydroxyethyl starch (HES 200/0.5), or modified Ringer's lactate for fluid resuscitation | intensive insulin therapy placed critically ill patients with sepsis at increased risk for serious adverse events related to hypoglycemia. In this study, HES was harmful, and its toxicity increased with accumulating doses | **9** |
|  | tumor necrosis factor receptor: Fc fusion protein | septic shock patients | a single intravenous infusion of one of three doses of TNFR:Fc (0.15, 0.45, or 1.5 mg per kilogram of body weight) or placebo | treatment with the TNFR:Fc fusion protein does not reduce mortality, and higher doses appear to be associated with increased mortality | **14** |
|  | recombinant human interleukin 1 receptor antagonist (rhIL-1ra) | patients with sepsis syndrome | a continuous 72-hour intravenous infusion of rhIL-1ra (1.0 or 2.0 mg/kg per hour) or placebo | rhIL-1ra treatment did not increase survival time compared with placebo. Treatment with rhIL-1ra results in a dose-related increase in survival time among patients with sepsis who have organ dysfunction and/or a predicted risk of mortality of 24% or greater | **17** |
|  | anti—tumor necrosis factor α monoclonal antibody (TNF-α MAb) | patients with sepsis syndrome | Patients received a single infusion of 15 mg/kg of TNF-α MAb, 7.5 mg/kg of TNF-α MAb, or placebo. | no decrease in mortality between placebo and TNF-α MAb in all infused patients | **24** |
| Negative | High dose of methylprednisolone or dexamethasone | Severe septic shock patient | 59 patients randomly assigned to a methylprednisolone, dexamethasone, or control group | corticosteroids do not improve the overall survival of patients with severe, late septic shock but may be helpful early in the course and in certain subgroups of patients. | **29** |
|  | 50 mg of intravenous hydrocortisone | Septic shock patient | 50-mg intravenous bolus  every 6 hours for 5 days, then tapered to 50 mg  intravenously every 12 hours for days 6 to 8, 50 mg  every 24 hours for days 9 to 11, and then stopped | Hydrocortisone did not improve survival or reversal of shock in patients with septic  shock | **31** |
|  | stress doses of hydrocortisone | patients who met the ACCP/SCCM criteria for septic shock | Hydrocortisone was started with a loading dose of 100 mg given within 30 mins and followed by a continuous infusion of 0.18 mg/kg/hr. | Overall shock reversal and mortality were not significantly different between the groups | **36** |
|  | nitric oxide synthase inhibitor NG monomethyl-L-arginine (L-NMMA) | severe sepsis associated with hypotension | Measurements of haemodynamic, haematological, and biochemical variables were made after intravenous dose of L-NMMA | Low doses of L-NMMA cause a widespread increase in vascular tone and raise blood pressure in patients with septic shock. | **42** |
|  | drotrecogin  alfa (activated) (DrotAA) | patients with severe sepsis and a low risk of death | intravenous infusion of placebo or DrotAA (24ug per kilo-gram of body weight per hour) for 96 hours | The absence of a beneficial treatment effect, coupled with an increased incidence of  serious bleeding complications, indicates that DrotAA should not be used in patients  with severe sepsis who are at low risk for death | **44** |
|  | Tifacogin (recombinant TFPI) | patients with severe sepsis | 0.025 mg/kg per hour of tifacogin as a c ontinuous intravenous infusion for 96 hours or an equivalent volume of placebo | Treatment with tifacogin had no effect on all-cause mortality in pa-tients with severe sepsis and high INR | **45** |
